# Social Media Participation, Low-Carbon Agricultural Practices, and Economic Performance of Banana Farmers in Southern China

**DOI:** 10.3389/fpsyg.2021.790808

**Published:** 2021-12-17

**Authors:** Qi Yang, Yueji Zhu, Fang Wang

**Affiliations:** Management School, Hainan University, Haikou, China

**Keywords:** low-carbon agricultural practices, social media participation, endogenous-treatment Poisson regression, quantile regression, economic performance

## Abstract

Low-carbon agricultural practices (LAPs) can reduce carbon emissions in agricultural production for farmers in developing countries. However, the role of emerging social media has not received enough attention in the diffusion of LAPs among farmers. This study first attempts to examine the impact of farmers’ social media participation on their adoption intensity of LAPs using the Zero-truncated Poisson model and specify the effect of each participation activity on social media by the endogenous-treatment Poisson regression model, then discuss the economic performance of LAPs using the quantile regression model, based on the primary data collected from banana farmers in Southern China. The results show that social media participation exerts a positive and significant effect on farmers’ adoption intensity of LAPs. Specifically, the adoption intensity of LAPs in the treated group who participated in the short-video social media is about 1.1 times higher than that in the control group. The treatment effects of the five activities (watch, like, forward, comment, and release) on farmers’ adoption intensity of LAPs are positive and significant. We also find that adoption of LAPs can increase household income of farmers, and the effect presents particularly significant for those at the higher income level. Whilst, Social media participation can significantly increase household income of farmers who are at the lower income level. Our findings underscore the important role of social media in the diffusion of LAPs among farmers and income growth of households in developing countries. Thus, supportive strategies can be designed by policymakers for encouraging farmers to participate the emerging social media platforms and adopt more LAPs in agricultural production.

## Introduction

Climate change seriously threatens the global environment and food security of human society (Pachauri et al., 2014; Tubiello et al., 2015; Marino et al., 2016; Guo et al., 2021). According to the report of IPCC, greenhouse gas emissions from agricultural production in the food system account for 16–27%, and emissions from transport, packaging, processing and loss and waste account for 5–10%, so the global food system accounts for 21–37% of man-made greenhouse gas emissions (Mbow et al., 2019; Xu et al., 2020). Coordinated approaches to reducing greenhouse gas emissions are urgently needed (Ionescu, 2020). To reduce agricultural carbon emissions, it requires a transformation from “high-carbon” agriculture to “low-carbon” agriculture. It is believed that the application of low-carbon agricultural practices (LAPs) is an important action to achieve the goal of agricultural green production (Liu et al., 2020; He et al., 2021; Li et al., 2021). Farmers’ adoption of LAPs is critical for promoting the development of green agriculture.

Existing studies have found that LAPs exerted positive impact on agricultural productivity and improved low-carbon efficiency at the same time (Ding et al., 2009; Abdulai and Huffman, 2014; Pfeiffer and Lin, 2014; Ismael et al., 2018; Midingoyi et al., 2019; He et al., 2021). However, the LAPs adoption rate is still low in rural areas of developing countries (Adolwa et al., 2019; Midingoyi et al., 2019; Ma and Wang, 2020), and previous studies documented that farmers’ decision of adopting agricultural management practices were influenced by various factors, such as household characteristics, plot-specifics and institutional factors (Defrancesco et al., 2008; Maroušek, 2013; Ghimire and Huang, 2016; Suvedi et al., 2017; Dalemans et al., 2019; Liu et al., 2019). In addition, a variety of studies have explored the impact of social network on farmers’ adoption of innovative agricultural technologies (Bandiera and Rasul, 2006; Magnan et al., 2015; Li et al., 2020). Some found that social learning among farmers’ social network gradually increased the adoption of resource-conservation technologies (Magnan et al., 2015). Li et al. (2020) also suggested that cooperative membership showed a positive sign on farmers’ adoption of low-carbon agricultural technologies in the case of rice production system. Others argued that the knowledge or information about innovative technologies gained from social network may be asymmetric and limited (Miroslava et al., 2020). Recent studies indicated that insufficient knowledge may hinder the diffusion of innovative agricultural technologies (Pan et al., 2017; Ma and Wang, 2020). As Internet use increases in rural areas, scholars discussed the positive relationship between information and communication technologies (ICT) usage and farmers’ adoption of innovations (e.g., Aker et al., 2016; Salim et al., 2016; Ma et al., 2018b; Ma and Wang, 2020). Nevertheless, little is known about the role of emerging short-video social media in farmers’ adoption of LAPs in agricultural practices so far.

As short-video social media apps (e.g. Tiktok) are featured by easy to entry, condensed video content and extensive social interactions (watch, like, forward, comment and release), farmers can participate in it easily with a smartphone (Liu, 2020). Compared with the traditional text and picture forms, the information delivered by short-videos in dynamic forms is more vivid, understandable and acceptable for farmers who usually do not have enough education in developing regions. In China, the number of Internet users has reached 1.011 billion, of which 297 million are in rural areas, and the number of short-video users has reached 888 million, accounting for 87.8% of Internet users (CNNIC, 2021). On the one hand, through the novel and popular social media, farmer can easily access to these short videos that can provide agriculture-related information and knowledge. It may affect farmers’ decision-makings in agricultural production. On the other hand, participation in the short-video social media can somehow expand farmers’ social network. It breaks the barriers of geographic distance for social interactions among farmers from different areas and makes the information disseminated in an efficient way. Thus, it leads to diffusion of agricultural innovations among farmers.

We attempt to contribute to the existing literature concerning farmers’ adoption of LAPs from following aspects. First, the association between farmers’ participation in short-video social media and adoption of LAPs has been rarely explored, though previous studies have investigated the role of smartphone use or Internet use in farmers’ decisions of adopting innovations. Thus, the first objective of this study is to evaluate the impact of farmers’ participation in the emerging social media on their adoption intensity of LAPs. More specifically, we discuss the effects of five activities of participation in short-video social media on farmers’ use of LAPs. Second, this study is to examine the heterogenous effects of social media participation and adoption intensity of LAPs on farmers’ income, using the quantile regression model. Empirical results from least squares estimation can only show the average effect, while the quantile regression can estimate the variation that occur around the mean of the independent variable, and detect the effects across the entire quantile distribution of the independent variable (Rodriguez-Caro et al., 2016). Third, considering that farmers’ participation in the short-video social media is not a random behavior, we employ the endogenous-treatment Poisson regression model to control the selection bias of sample farmers whose decisions may be affected by some observable and unobservable factors.

The remainder of this paper is organized as following: Section “Empirical Strategies” introduces the empirical strategies; the data collection and descriptive statistics are presented in Section “Data and Descriptive Statistics”; the empirical results are given in “Results”; the effects of LAPs adoption intensity and social media participation on farmers’ income are discussed in Section “Economic Performance”; the last section presents the conclusions and policy implications.

## Empirical Strategies

This paper aims to identify the role of social media participation on farmers’ adoption intensity of LAPs and clarify the heterogenous effect of adoption intensity on farmers’ income in Southern China. Firstly, we use Zero-truncated Poisson to evaluate the impact of social media participation on farmers’ adoption intensity of LAPs. Then, we model the counterfactual scenarios of five specific activities of social media participation on farmers’ adoption intensity of LAPs through the endogenous-treatment Poisson regression model. Finally, the quantile regression model is used to estimate the heterogenous effects of adoption intensity and social media participation on farmers’ income.

### Zero-Truncated Poisson Model

Existing studies have indicated that the standard Poisson regression was often designed for identifying the adoption intensity of agricultural innovative technologies (Winkelmann, 2008; Cameron and Trivedi, 2013; Ehiakpor et al., 2020). However, the basic requirement of standard Poisson regression model is that the conditional expectation should be equal to the conditional variance, that is equi-dispersion, *E*(*y*|μ) = *V*(*y*|μ) = μ. Unfortunately, this is not always the case. When the conditional variance is greater than the conditional expectation, it is called over-dispersion. While in the case where the conditional variance is smaller than the conditional expectation, it is called under-dispersion. In addition, there are cases of excess zeros in the count data set. Thus, if we do not take the type of count data into account, the estimation of standard Poisson regression may be inaccurate (Greene, 2002; Erdman et al., 2008; Ehiakpor et al., 2020).

However, we check the sample data used in this study and find that each farmer adopted at least two LAPs, that’s to say the sample data is characterized by non-zero outcomes in this study. Without considering the data characteristic of zero truncation, it may lead to a biased estimation (Lord et al., 2005). Therefore, a zero-truncated Poisson model can be used to correct the bias caused by non-zero outcomes (Ehiakpor et al., 2020).

Following Long and Freese (2014), the zero-truncated Poisson model is a modification based on the standard Poisson regression model and it originates from the standard Poisson regression model:


(1) $$\mathrm{Pr}\left(y_{j}=k|x_{j}\right)=\frac{\text{exp}\,\left(-\mu_{j}\right)\,\mu_{j}^{y_{j}}}{y_{j}}$$

where for a given *x*_*j*_, the likelihood of observing zero outcomes is *Pr*⁡(*y*_*j*_ = 0|*x*_*j*_) = exp(−μ_*j*_), while the likelihood of observing non-zero outcomes is denoted as Pr(yj>0|xj)=1-exp(-μ)j.

The conditional probability equation can be expressed as:


(2) $$\mathrm{Pr}\left(A|B\right)=\frac{\text{Pr}\,\left(A\,B\right)}{\text{Pr}\,\left(B\right)}$$

When we observed a specific count data, such as *y*_*j*_ = *k*, and given that *k* is a value of non-zero, then the conditional probability equation could be specified as follows:


(3) $$\mathrm{Pr}\left(y_{j}=k\,|y_{j}>\,0,x_{j}\right)=\frac{\text{Pr}\left(y_{j}=k\&y_{j}>0|x_{j}\right)}{\text{Pr}\left(y_{j}>0|x_{j}\right)}$$

In empirical equation, the zero-truncated Poisson model also can be defined as:


(4) $$\mathrm{Pr}\left(y_{j}>0|x_{j}\right)=\beta \,x_{j}+\mu_{j}$$

Where *x*_*j*_ is the independent variables, β is the parameter to be estimated, and μ_*j*_ denotes the error term.

### Endogenous-Treatment Poisson Regression Model

To explore impact of farmers’ participation in the short-video social media on farmers’ adoption intensity of LAPs, we need consider that farmers’ participations are not random choices in our sample, and some unobservable factors (e.g., farmers’ intrinsic capacities) may affect their choices. Thus, social media participation of farmers is considered as an endogenous variable *P*_*j*_.

In previous studies, scholars relied on the use of instrumental variable (IV), propensity scores matching (PSM) approach and inverse-probability weighted regression adjusted (IPWRA) estimator to address the issue of selection bias (Kassie et al., 2011; Shiferaw et al., 2014; Manda et al., 2018; Tambo and Mockshell, 2018; Adane et al., 2019; Danso-Abbeam and Baiyegunhi, 2019; Fentie and Beyene, 2019; Hou et al., 2019). While the PSM could only correct the selection bias caused by observable confounding factors, the IV and IPWRA estimator account for the heterogeneity caused by both observable and unobservable confounding factors. Considering that the outcome variable is a count data, we follow Miranda (2004) to implement endogenous-treatment Poisson regression model to address the issue of sample selection bias. However, the PSM approach and IPWRA are used for the robustness check in this study.

In the endogenous-treatment Poisson regression model, farmers’ decision of adopting a number of LAPs or none can be denoted as *L*_*j*_ = *k*(*k* = 0,1,2,…,8). We can define a dummy *S*_*j*_ that represents a sample selection rule, and *S*_*j*_ = 1 if *L*_*j*_ is observed as a non-zero outcome, while *S*_*j*_ = 0 if farmers adopted none of the LAPs (*L*_*j*_ is missing). The endogenous treatment and selection dummies can be generated from the continuous unobserved latent variables that indicates the potential satisfaction obtained from participating in each activity on the short-video social media and adopting any of LAPs:


(5) $$P_{j}^{{}^{*}}=\alpha \,Z_{j}^{\prime }+\varepsilon_{j}$$

(6) $$S_{j}^{{}^{*}}=\delta \,X_{j}^{\prime }+\gamma \,P_{j}+\varphi_{j}$$

With *P*_*j*_ = 1 if $P_{j}^{{}^{*}}>0$, and *S*_*j*_ = 1 if $S_{j}^{{}^{*}}>0$.

As the outcome equation in endogenous-treatment Poisson regression model follows a Poisson distribution, it can be specified as:


(7) $$L_{j}=\left\{ \begin{array}{c} 0\;i\,f\,S=0\\ \frac{\left[\varepsilon^{L_{j}}\,\exp\left(-\varepsilon \right)\right]}{L_{j}!}\;i\,f\,S=1 \end{array}\right.$$

(8) $$E\,\left(L_{j}|X_{j},P_{j},\varphi_{j}\right)=\text{exp}\,\left(\delta \,X_{j}+\gamma \,P_{j}+\varphi_{j}\right)$$

While $Z_{j}^{\prime }$ is vector of the covariates in the binary treatment equation, $X_{j}^{\prime }$ is vector of covariates used in the count outcome equation, ε_*j*_ and φ_*j*_ are error terms for the treatment and the outcome equation, respectively.

This model can measure the average treatment effect of the treated group (ATT). The ATT is defined as the average difference between the potential outcome of treated group and its counterfactual context (Lokshin and Sajaia, 2004; Imbens and Wooldridge, 2009; Takahashi and Barrett, 2013; Gokul et al., 2019; Ma and Wang, 2020). Using the estimated results of endogenous-treatment Poisson regression model, the ATT can be specified as:


(9) $$ATT=E\left(L_{1\,j}-L_{0\,j}|P_{j}=1\right)=E\left(L_{1\,j}|P_{j}=1\right)-E\left(L_{0\,j}|P_{j}=1\right)$$

where E(⋅) represents the expectation operator, *L*_*1j*_ is the potential outcome for farmers who participate in social media, *L*_*0j*_ is the potential outcome for farmers in the counterfactual context.

### Quantile Regression Model

The traditional OLS regression can only provide an average effect in estimation and it may be simply affected by the outliers, while the quantile regression model proposed by Koenker and Bassett (1978) uses the weighted average of the absolute value of the residual as the objective function of minimization, and it is not easily affected by the outliers. The quantile regression model provides a more comprehensive information about conditional distributions (Rodriguez-Caro et al., 2016). The quantile regression model can supply estimated result in every quantile, and the results can be more accurate. Moreover, the estimations of the quantile regression model are more robust than that in OLS regression (Xu and Lin, 2018).

The empirical form of quantile regression model is given as:


(10) $$I_{j}=\theta_{\tau }\,x_{j}^{\prime }+\omega_{\tau \,j},0<\tau <1$$

(11) $$Q_{\tau }\,\left(I_{j}|x_{j}\right)=\theta_{\tau }\,x_{j}$$

Where $x_{j}^{\prime }$ is the vector of explanatory variables, *I*_*j*_ indicates the explained variable (farmer’s income), ω_*τj*_ is the random error term, *Q*_τ_(*I*_*j*_|*x*_*j*_) is the τth quantile of the explained variable. θ_τ_ is the coefficient of τth quantile, and its regression estimator ${\hat{\theta }}_{\tau }$ can be defined by the following minimization equation:


(12) $$\min_{\theta_{\tau }}\sum_{j:I_{j}\geq \theta_{\tau }\,x_{j}^{\prime }}^{n}\tau \,\left|I_{j}-\theta_{\tau }\,x_{j}^{\prime }\right|+\sum_{j:I_{j}<\theta_{\tau }\,x_{j}^{\prime }}^{n}\left(1-\tau \right)\,\left|I_{j}-\theta_{\tau }\,x_{j}^{\prime }\right|$$

when the value of τ is different, it can obtain different parameter estimates. If τ is equal to 0.5, it is a median regression, also known as Least Absolute Deviation estimator.

To investigate the connection between farmers’ adoption intensity of LAPs and income effectively, we chose several representative quantiles, such as 25th, 50th, 75th quantiles. And we applied the bootstrap method to calculate the standard error in quantile regression. Unlike the traditional segmental regression, the parameters of different quantiles can be estimated by using all the sample data in the quantile regression model (Cade and Noon, 2003; Xu and Lin, 2018).

## Data and Descriptive Statistics

### Data Collection

The data used in this study was collected from the field survey of banana farmers in 2021. According to the data from China’s National Banana Industry Technology System (CNBITS), the total output of banana was 5.348 million tons, and Hainan province was ranked as the third-largest producing area in China with a total output of 1.084 million tons in 2020. First, we purposely selected three counties in Hainan province, including Chengmai, Lingao, and Changjiang. The three counties are the main banana farming areas in this province. Second, two to four townships were randomly selected according to the size of banana cultivation in each county. Then we randomly chose several villages from each township, and around 15–25 banana farmers were selected and interviewed in each village. A total of 307 banana farmers participated in the survey. Finally, we obtained 282 valid respondents. The data was collected by face-to-face interviews based on a structured questionnaire for this study. Respondents were informed about the purpose before the start of the questionnaire survey. The respondents acknowledged that the data of the questionnaire survey would be used only for academic research purposes. We ensured that the privacy of the respondents and their households would not be compromised, and all the data was collected and used based on the voluntary participation of the respondents. Verbal consent was also taken from them. Table 1 presents the sample distribution.

**TABLE 1:** Description of sample distribution.

County	Observations	Percentage (%)
Chengmai	84	29.79
Lingao	96	34.04
Changjiang	102	36.17

### Variable Measurement

LAPs are characterized by the features of seeking to mitigate carbon emissions and protecting the environment without harming the economic well-being of farmers (Solazzo et al., 2016; Yue et al., 2017; Zheng et al., 2017; Liu et al., 2019; Li et al., 2020). However, there is no unified classification of LAPs. Based on the LAPs used in banana cultivation, this study specifically considers eight management practices including zero or minimum tillage, fallow, intercropping, soil testing, organic fertilizer, biopesticide, drip fertigation and crop residue retention.

The main explained variable is a count variable *Adoption intensity* in this study. It is defined as the number of LAPs adopted by a banana farmer, and its value ranges from 0 to 8. Following previous studies (Kassie et al., 2013; Manda et al., 2016; Liu et al., 2019; Ma and Wang, 2020), we asked farmers whether they used the practices from a list of eight LAPs during the latest growing season, then we calculate the number of LAPs adopted by each farmer.

Social media participation is an important explanatory variable that ranges from 0 to 5, and it is defined as the degree of farmers’ participations in the short-video social media. Specifically, there are five activities of the participation, including watching, liking, forwarding, commenting and releasing short-videos. And each activity can be indicated by a binary variable. It equals 1 if a farmer takes a given activity, otherwise 0.

In this study, we also include farmers’ socioeconomic characteristics as the control variables, including individual characteristics, farmer household resources, agricultural conditions, farmers’ awareness of climate change, and other variables. The definition and measurement of the variables are given in Table 2.

**TABLE 2:** Definition, measurement and descriptive statistics of the variables.

Variables	Definition and measurement	Mean	SD
**Dependent variables**			
Adoption intensity	Numbers of Low-carbon agricultural practices (LAPs) used in banana cultivation (from 0 to 8)	4.46	1.19
Banana income	Total income from banana cultivation (10,000 CNY)	7.04	9.81
**Independent variable**			
Watch	= 1 if the respondent watches agriculture-related short-videos; otherwise = 0	0.54	0.50
Like	= 1 if the respondent likes agriculture-related short-videos; otherwise = 0	0.42	0.49
Forward	= 1 if the respondent forward agriculture-related short-videos; otherwise = 0	0.30	0.46
Comment	= 1 if the respondent makes comment on agriculture-related short-videos; otherwise = 0	0.27	0.44
Release	= 1 if the respondent release agriculture-related short-videos; otherwise = 0	0.24	0.43
Social media participation	Degree of the respondent’s participations in the short-video social media (from 0 to 5)	1.77	1.83
Age	Age of the respondent	47.59	11.02
Gender	= 1 if the respondent is male; otherwise = 0	0.80	0.40
Education	Education years of the respondent	8.00	3.44
Marriage	= 1 if the respondent has a spouse; otherwise = 0	0.94	0.24
Party membership	= 1 if the respondent is a member of the Communist Party of China; otherwise = 0	0.21	0.41
Religion	= 1 if the respondent has a religion; otherwise = 0	0.06	0.23
Risk preference	= 1 if the respondent prefers things with certainty; otherwise = 0	0.27	0.44
Personality	= 1 if the respondent can be the first one to adopt innovations; = 2 if the respondent adopts innovations when others have adopted; = 3 if the respondent adopts innovations only when they see benefits	2.26	0.91
Off-farm work	= 1 if the respondent undertakes an off-farm work; otherwise = 0	0.48	0.50
Farming experience	Years of engaging in agricultural activities	25.40	12.54
Household income	Total household income (10,000 CNY)	11.98	14.12
Labor	Numbers of family’s labor force in agriculture	2.72	1.37
Children	Numbers of family’s children in school	1.43	1.27
Farm size	Farm size of banana (mu*^a^*)	12.41	13.61
Land tenure stability	= 1 if the respondent owns farmland area more than 50% of the total cultivated area in banana cultivation; otherwise = 0	0.61	0.49
Level farmland	= 1 if the farmland of banana cultivation is level; otherwise = 0	0.76	0.43
Irrigation status	= 1 if the farmland of banana cultivation with good irrigation condition; otherwise = 0	0.51	0.50
Soil degradation	= 1 if the respondent perceives that the land has deteriorated; otherwise = 0	0.41	0.49
Threat perception	= 1 if the respondent is aware of the threat of climate change to agriculture; otherwise = 0	0.86	0.35
Loan	= 1 if the household takes loan in 2021; otherwise =0	0.43	0.50
Cooperative membership	= 1 if the respondent is a member of agricultural cooperatives; otherwise = 0	0.08	0.27
Training	= 1 if the respondent takes any training about banana cultivation in 2021; otherwise = 0	0.32	0.47
Distance to bus station	Distance to the nearest bus station (km)	5.74	7.62
Social ties	Numbers of close connected friends	17.38	24.88

*^a^1 mu = 1/15 hectare.*

### Descriptive Statistics

The summary of the statistics of variables are presented in Table 2. The mean degree of the respondent’s participation in social media of agricultural short-video is 1.77. More specifically, 54% of the respondents watched agriculture-related short-videos through the social media and 42% of them liked agriculture-related short-videos. Farmers who forwarded agriculture-related short-videos account for 30% in this study. While 27% and 24% of the interviewed farmers commented on and released agriculture-related short-videos, respectively. The male respondents accounts for 80%, because they are usually the household heads and make decisions in agricultural production in rural China. The respondents are 48 years old on average and have an about 8 years of education. The average household income is 11.98 (ten thousand yuan), and a family has 2.72 labors engaged in agriculture and 1.43 children on average. The average banana farm size is about 12.41 mu (0.83 hectare). This implies most of the banana farm households are smallholders in this study. As to the awareness of climate change, the majority of farmers perceived the threat of climate change to agriculture and 41% of them agree that soil fertility has deteriorated seriously. The average distance from their residents to bus station is reported as 5.74 km. And the farmers reported that they have about 17.38 social ties. It means these farmers have frequent social interactions with each other.

Table 3 shows the descriptive statistics of LAPs. Of the 282 sampled banana farm households, about 35% adopted zero or minimum tillage practice and nearly half (49%) fallowed. Only 26% banana farmers used intercropping and 14% of them adopted soil testing for fertilization of their banana plots. The statistics also reveals that almost 99% of banana farmers used organic fertilizer. However, the adoption rate of biopesticide is approximately 38%. There are about 90% of the respondents who adopted drip fertigation in banana cultivation and 95% of the respondents who practiced crop residue retention.

**TABLE 3:** Descriptive statistics of LAPs.

Components	Description	Mean	SD
Zero or minimum tillage	= 1 if adopted; otherwise = 0	0.35	0.48
Fallow	= 1 if adopted; otherwise = 0	0.49	0.50
Intercropping	= 1 if adopted; otherwise = 0	0.26	0.44
Soil testing	= 1 if adopted; otherwise = 0	0.14	0.35
Organic fertilizer	= 1 if adopted; otherwise = 0	0.99	0.10
Biopesticide	= 1 if adopted; otherwise = 0	0.38	0.49
Drip fertigation	= 1 if adopted; otherwise = 0	0.90	0.30
Crop residue retention	= 1 if adopted; otherwise = 0	0.95	0.21

**FIGURE 1:**
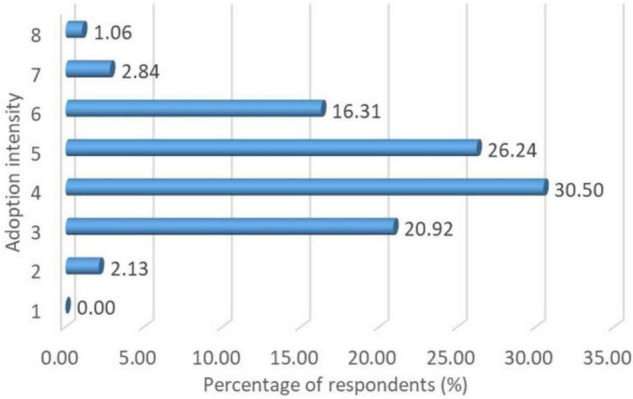
Distribution of adoption intensity of LAPs.

The distribution of adoption intensity of LAPs is shown in Figure 1. The value of adoption intensity actually ranges from 2 to 8, as each banana farmer has adopted at least two LAPs according to our survey. The mean number of LAPs adopted is about 4.46 (Table 2). Figure 1 also reveals that 2.30% adopted two LAPs, while the majority (93.97%) of the banana farmers adopted 3–6 LAPs. About 3.90% of banana farmers used 7–8 LAPs in the study area.

## Results

### Impact of Social Media Participation on Farmers’ Adoption Intensity of Low-Carbon Agricultural Practices

The estimated results of the impact of social media participation on farmers’ adoption intensity of LAPs are presented in Table 4. Model 1 only includes the main explanatory variable s*ocial media participation*, while Model 2 adds all the control variables based on Model 1. Both the coefficients of social media participation show significant and positive signs. Social media participation can enlarge the scope of farmers’ social interactions, while their social networks are positively associated with the adoption of new agricultural practices or technologies (Bandiera and Rasul, 2006; Conley and Udry, 2010; Nakano et al., 2018; Zhu et al., 2019; Yang et al., 2020). The estimated results show that the more active the farmers participate in the short-video social media, the more LAPs they adopt in banana cultivation. In addition, the marginal effect shows a significant and positive sign, indicating that for every 1% increase in social media participation, the likelihood of farmers’ adoption intensity could increase by 12.70%. This may be because farmers can obtain more information about the economic and environmental benefits of LAPs in long-term through the short-video social media. It may broaden their social networks, so they can learn and imitate from the video bloggers who have better knowledge of LAPs.

**TABLE 4:** The impact of social media participation on farmers’ adoption intensity of LAPs.

Variables	Model 1	Model 2	Marginal effects
Social media participation	0.041 (0.009)***	0.029 (0.010)***	0.127 (0.043)***
Age		0.003 (0.002)**	0.015 (0.007)**
Gender		0.055 (0.045)	0.241 (0.198)
Education		−0.005 (0.005)	−0.021 (0.024)
Marriage		0.082 (0.081)	0.361 (0.355)
Party membership		0.008 (0.041)	0.037 (0.181)
Religion		0.094 (0.061)	0.414 (0.269)
Risk preference		0.046 (0.036)	0.204 (0.160)
Personality		0.035 (0.018)*	0.153 (0.078)*
Off−farm work		0.125 (0.034)***	0.550 (0.149)***
Farming experience		−0.003 (0.002)*	−0.012 (0.007) *
Household income		0.001 (0.001)	0.005 (0.006)
Labor force		0.002 (0.010)	0.011 (0.044)
Children		−0.004 (0.012)	−0.017 (0.054)
Farm size		0.000 (0.001)	0.001 (0.007)
Land tenure stability		0.040 (0.035)	0.177 (0.154)
Level farmland		0.064 (0.039)	0.282 (0.173)
Irrigation status		0.048 (0.031)	0.211 (0.137)
Soil degradation		0.077 (0.031)**	0.339 (0.136)**
Threat perception		0.093 (0.049)*	0.409 (0.217)*
Loan		−0.017 (0.032)	−0.076 (0.142)
Cooperative membership		0.077 (0.046)*	0.341 (0.201)*
Training		−0.002 (0.037)	−0.008 (0.164)
Distance to bus station		−0.004 (0.002)*	−0.017 (0.010)*
Social ties		−0.001 (0.001)	−0.003 (0.003)
Constant	1.408 (0.023)***	0.902 (0.049)***	
Observations	282	282	
Wald chi2	21.36	109.85	
Prob>chi2	0.000	0.000	
Pseudo R2	0.007	0.021	
Log pseudolikelihood	−508.660	−487.268	

Standard errors are in parentheses, and ***, **, * represent significance level at 1, 5, 10%, respectively.

In terms of the control variables, the significant and positive effect of farmers’ age on farmers’ adoption intensity of LAPs implies that older farmers have a higher possibility of adopting more LAPs than younger farmers. The older farmers may experience more environmental hazards and be more aware of the potential loss brought by climatic shocks, so they would pay more attention to the long-term benefits of agricultural practices than the younger counterparts, and adopt more LAPs. This result is in line with the findings of Tesfaye and Tirivayi (2018) and Ehiakpor et al. (2020). Farming experience shows a significant but negative sign, suggesting that farmers with more farming experience adopt less LAPs. This is probably because the experienced farmers are more confident in the traditional farming practices. This result contradicts the findings of Ehiakpor et al. (2020), who argued that experienced maize farmers were more likely to practice the LAPs. The results also show that the farmers, who adopt innovations only if they see the benefits, would be inclined to enhance their adoption intensity of LAPs. Smallholder farmers are mostly risk-aversion actors. They adopt LAPs only if they can perceive the advantages of LAPs. In addition, off-farm work significantly and positively affects farmers’ adoption intensity of LAPs. Farmers who are engaged in off-farm work are more open-minded and easily exposed to innovations, thus they are more capable of using more LAPs than farmers who are not engaged in off-farm work. This finding is consistent to that of Yahaya (2015), but contrary to that of Ahmed (2015); Manda et al. (2016), and Ehiakpor et al. (2020).

Both soil degradation and farmers’ perceived threat of climate change have significant and positive impacts on their adoption intensity of LAPs. It indicates that farmers with poorer land or more awareness of climate change have higher tendency to use LAPs for sustainable outputs. Meanwhile, a significant and positive effect of cooperative membership is identified on farmers’ adoption intensity of LAPs. Farmers who are members of agricultural cooperatives have higher likelihood of adopting LAPs compared with those who are not. This is consistent with earlier studies of Ma et al. (2018a) and Ma and Wang (2020). Also, we observe a significant negative association between distance to bus station and farmers’ adoption intensity of LAPs, indicating that households closer to the bus station are more inclined to adopt more LAPs compared with those far from the bus station. Farmers who are closer to bus station can easily access the market of agricultural inputs, and it is more convenient for them to gain the information about LAPs.

### Treatment Effects of Five Activities of Participation in Social Media

The comparison of mean value is used to illustrate the ratio of potential outcome of the treated group to that of the control group. As shown in Table 5, the coefficient in the second row suggests that the mean potential outcome of the treated group who watched agriculture-related short-videos is about 1.06 times higher than that of the control group who did not watch agriculture-related short-videos. In other words, the average number of LAPs adopted in the treated group is significantly more than the average number of LAPs used in the control group. The coefficients in other rows of Table 5 show effects in the same way. Thus, the estimated results show that the average number of LAPs adopted by farmers who participated in the short-video social media is higher than those who did not participate in social media. However, a simple comparison of mean values of the two groups can still be misleading without controlling systematic differences between participants and non-participants (Danso-Abbeam and Baiyegunhi, 2019). Therefore, we estimate the ATT to identify the mean difference between the actual expected value and the counterfactual expected value of farmers in the endogenous-treatment Poisson regression model.

**TABLE 5:** The ratio of adoption intensity in the treated group to that in the control group.

	Coef.	*Z*-value
Watch	1.063 (0.036)*	1.80
Like	1.077 (0.388)**	2.07
Forward	1.110 (0.040)***	2.93
Comment	1.100 (0.041)**	2.53
Release	1.080 (0.042)**	2.01

Standard errors are in parentheses, and ***, **, * represent significance level at 1, 5, 10%, respectively.

As shown in Table 6, the ATT of watching agriculture-related short-videos on farmers’ adoption intensity of LAPs is about 0.28 with a statistically significance at 10% level. Farmers who watched agriculture-related short-videos adopted more LAPs than those who did not watch agriculture-related short-videos. Liking activity of farmers on the short-video social media can increase their adoption intensity of LAPs by 34%. Similarly, forwarding and commenting activity can increase farmers’ adoption intensity of LAPs by 45–49%. Releasing agriculture-related short-videos on the social media can promote farmers to use 40% more LAPs. In summary, farmers who participated in the short-video social media are observed a higher adoption intensity of LAPs than those who did not participate in the social media.

**TABLE 6:** Treatment effects of social media participations on farmers’ adoption intensity of LAPs.

	ATT	*Z*-value
Watch	0.276 (0.152)*	1.81
Like	0.341 (0.164)**	2.08
Forward	0.487 (0.168)***	2.90
Comment	0.447 (0.178)**	2.52
Release	0.359 (0.183)**	1.97

Standard errors are in parentheses, and ***, **, * represent significance level at 1, 5, 10%, respectively.

### Robustness

The robustness of estimated results is checked using the PSM approach and IPWRA estimator in this study. As shown in Table 7, the results suggest that the effects of five activities (watch, like, forward, comment and release) through the social media on farmers’ adoption intensity of LAPs are consistent with our previous estimated results. The average treatment effect of watching activity on farmers’ adoption intensity are about 0.13–0.20 with a positive sign, although it is not statistically significant. Basically, the estimated results of the two estimation strategies confirm that the results of endogenous-treatment Poisson regression model are robust enough.

**TABLE 7:** Robustness check by using PSM approach and IPWRA estimator.

	ATT (PSM)	*Z*-value	ATE (IPWRA)	*Z*-value
Watch	0.128 (0.165)	0.77	0.197 (0.154)	1.27
Like	0.195 (0.238)	0.82	0.266 (0.161)*	1.65
Forward	0.475 (0.166)***	2.86	0.436 (0.145)***	3.00
Comment	1.053 (0.206)***	5.12	0.616 (0.178)***	3.46
Release	0.457 (0.201)**	2.28	0.349 (0.172)**	2.02

Standard errors are in parentheses, and ***, **, * represent significance level at 1, 5, 10%, respectively.

## Economic Performance

The long-term benefits can be achieved by using low-carbon practices in agricultural production (Teklewold et al., 2013; Kassie et al., 2015; Manda et al., 2016). To further understand the economic performance, we examine the effects of farmers’ adoption intensity of LAPs and social media participation on their incomes using the quantile regression model.

The marginal effects of the independent variables on different quantiles of banana income and household income are revealed in Table 8. As discussed before, we focus on the results estimated at the 25th, 50th and 75th quantiles, respectively. Specifically, the coefficient of adoption intensity on banana income in the 75th quantiles is the largest (0.31), while those of 25th and 50th quantiles are 0.14 and 0.18, though the coefficients are not statistically significant at these quantiles. Our findings are contrary to that of Ma and Wang (2020), who found that sustainable agricultural practices decreased their farm incomes. We argue that LAPs adoption can generally lead to an increase in the income of banana farmers. The positive association has been also identified in existing studies. For example, Liang et al. (2021) found that adaptive agricultural practices, such as zero tillage and soil testing practices etc., increased the income of rice farmers in China. The adoption intensity of LAPs is positively and significantly associated with household income at the 75th quantiles, and the coefficient increases with the higher quantiles (0.31, 0.69, and 1.06). For low- and middle-income households, farmers are more dependent on traditional farming practices due to budget constraints. Thus, the correlation between adoption intensity of LAPs and household income is not statistically significant at the 25th and 50th quantiles. In contrast, farmers with high-income level can easily afford the new technologies, and are more likely to adopt more LAPs. In addition, some LAPs can reduce the effort of labor force in banana cultivation, thereby enabling farmers to participate in off-farm work. It can increase the household income of these farmers.

**TABLE 8:** Impact of social media participation and LAPs on farmers’ incomes.

	Banana income			Household income		
	τ = 25th	τ = 50th	τ = 75th	τ = 25th	τ = 50th	τ = 75th
Adoption intensity	0.140 (0.168)	0.182 (0.187)	0.307 (0.269)	0.306 (0.429)	0.685 (0.500)	1.060 (0.618)*
Social media participation	0.042 (0.108)	−0.076 (0.125)	−0.032 (0.200)	0.410 (0.246)*	0.156 (0.308)	0.406 (0.409)
Age	0.019 (0.027)	−0.010 (0.027)	−0.022 (0.036)	−0.011 (0.066)	0.031 (0.078)	0.089 (0.109)
Gender	0.169 (0.507)	0.108 (0.531)	−0.066 (0.650)	0.494 (1.064)	−1.070 (1.403)	−0.274 (1.749)
Education	−0.009 (0.057)	−0.053 (0.069)	0.023 (0.092)	0.036 (0.155)	−0.005 (0.189)	0.163 (0.252)
Marriage	0.117 (0.745)	0.197 (0.786)	0.143 (0.918)	0.098 (1.474)	0.790 (1.443)	1.874 (2.427)
Party membership	0.551 (0.436)	0.280 (0.504)	0.194 (0.678)	0.476 (1.206)	−0.601 (1.261)	−2.497 (1.804)
Religion	0.147 (0.741)	0.168 (0.922)	0.356 (1.967)	−0.018 (1.624)	1.351 (2.712)	2.794 (6.397)
Risk preference	0.398 (0.395)	0.232 (0.476)	−0.195 (0.635)	0.430 (0.919)	−0.933 (1.079)	−0.372 (1.537)
Personality	−0.303 (0.213)	−0.142 (0.233)	−0.078 (0.287)	−0.690 (0.426)*	−0.559 (0.541)	−1.484 (0.834)*
Off−farm work	−0.177 (0.425)	0.223 (0.420)	0.243 (0.586)	−0.173 (0.966)	−0.760 (1.162)	0.188 (1.474)
Farming experience	−0.009 (0.025)	−0.006 (0.026)	0.009 (0.035)	0.039 (0.051)	0.026 (0.066)	0.020 (0.097)
Labor force	0.004 (0.150)	0.123 (0.154)	−0.027 (0.216)	0.065 (0.267)	0.144 (0.336)	−0.038 (0.489)
Children	−0.113 (0.166)	−0.086 (0.153)	−0.129 (0.225)	−0.498 (0.355)	−0.797 (0.409)**	−0.915 (0.573)*
Farm size	0.317 (0.049)***	0.541 (0.074)***	0.667 (0.054)***	0.366 (0.103)***	0.637 (0.084)***	0.679 (0.099)***
Land tenure stability	−0.856 (0.477)*	−0.779 (0.561)	−1.310 (0.744)*	−2.150 (0.988)**	−0.495 (1.052)	0.589 (1.457)
Level farmland	0.106 (0.445)	−0.320 (0.515)	−0.343 (0.920)	−1.705 (1.199)	−2.120 (1.294)*	−0.622 (1.706)
Irrigation status	0.386 (0.337)	0.066 (0.388)	−0.453 (0.501)	0.509 (0.900)	−0.398 (1.071)	2.273 (1.576)
Soil degradation	−0.861 (0.339)***	−0.756 (0.413)*	−1.065 (0.615)*	−1.619 (0.882)*	−0.839 (0.996)	−1.280 (1.261)
Threat perception	0.046 (0.402)	−0.250 (0.413)	−0.003 (1.075)	−0.837 (1.205)	−0.315 (1.343)	−0.198 (2.076)
Loan	−0.240 (0.444)	−0.304 (0.420)	−0.387 (0.606)	−0.722 (0.852)	−0.660 (1.012)	0.339 (1.515)
Cooperative membership	0.661 (0.587)	0.730 (1.617)	1.978 (4.144)	1.910 (1.392)	2.007 (2.208)	4.097 (5.876)
Training	−0.331 (0.494)	0.324 (0.508)	0.868 (0.711)	−0.053 (1.026)	−0.508 (1.127)	−0.666 (1.647)
Distance to bus station	−0.020 (0.024)	−0.033 (0.028)	−0.045 (0.042)	−0.091 (0.055)*	−0.097 (0.066)*	0.021 (0.089)
Social ties	0.009 (0.007)	0.003 (0.006)	−0.004 (0.009)	−0.002 (0.018)	0.005 (0.028)	0.038 (0.047)
Constant	0.064 (1.548)	1.225 (1.848)	1.709 (2.266)	5.385 (3.847)	3.449 (4.737)	−2.567 (5.961)
Pseudo R2	0.304	0.424	0.552	0.236	0.291	0.355

Standard errors are in parentheses, and ***, **, * represent significance level at 1, 5, 10%, respectively.

Social media participation exerts a significant and positive impact on household income at 25th quantiles. Farmers who actively participate in social media may be more open-minded and more receptive to innovations. They are more likely to receive a higher household income. The association can be found in the impact of personality of farmers on their household income. The coefficients of farm size on banana income and household income are positive and statistically significant, indicating that farm size contributes to the increases in their incomes. These findings can also be seen in earlier studies (Kabunga et al., 2014; Ma and Wang, 2020). The significant negative association between land tenure stability and farmers’ incomes is presented in the results. Usually farmers with unstable land tenure rented more land for banana cultivation, so they can earn higher incomes. Farmers’ perception of soil degradation shows a negative and significant sign on banana income at all quantiles. Distance to bus station has a significant and negative sign on household income at 25th and 50th quantiles. It suggests that households closer to the bus station have more chance to earn higher household incomes.

**FIGURE 2:**
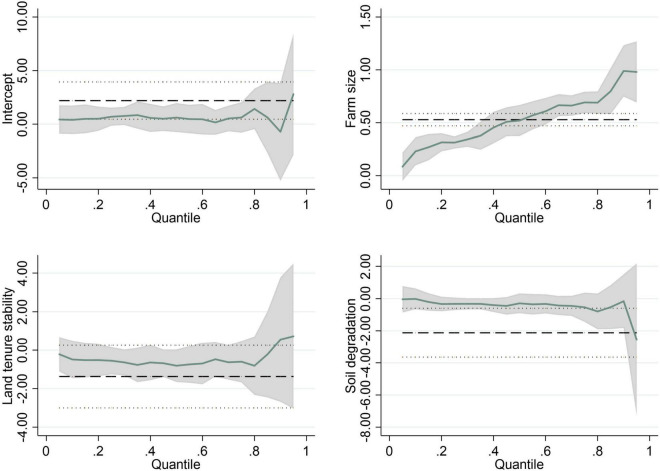
Quantile estimates: The impacts of factors on banana income. Shaded areas represent 95% confidence band for the quantile regression estimates. The black dotted lines denote the conventional 95% confidence intervals for the OLS.

**FIGURE 3:**
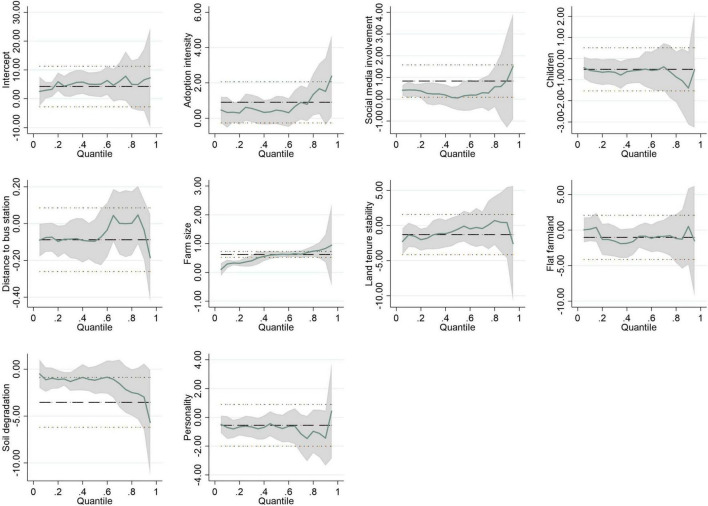
Quantile estimates: The impacts of factors on household income. Shaded areas represent 95% confidence band for the quantile regression estimates. The black dotted lines denote the conventional 95% confidence intervals for the OLS.

In order to illustrate the variation of the estimated coefficients with the quantile, we report the trends of the coefficients in Figures 2, 3. We only show the coefficient variation trends of the statistically significant variables in Table 8. The shape of the curve in the two Figures basically confirms the change of the estimated coefficients in Table 8. Moreover, the 95% confidence interval becomes wider at the right end of the conditional distribution, because the standard error of the estimated coefficient becomes larger. The quantile regression technique helps us to understand the comprehensive impacts of the driving forces on farmers’ incomes.

## Conclusion

The diffusion of LAPs among smallholder farmers has been widely deemed as an important strategy to reduce carbon emissions and promote sustainable agriculture in developing countries. Based on the primary data collected from banana farmers in Southern China, this study detects the role of the emerging social media in farmers’ adoption intensity of LAPs and discusses the average treatment effects of five specific activities of participation on farmers’ uptake of LAPs. Also, we explore the heterogenous effects of the LAPs and social media on farmers’ incomes using the quantile regression model.

We find that social media participation exerts a significant and positive impact on farmers’ adoption intensity of LAPs. Every 1% increase in social media participation could increase the likelihood of farmers’ adoption intensity by 12.70%. Besides, the results of endogenous-treatment Poisson regression model indicate that the five activities (watch, like, forward, comment and release) of social media participation have positive and significant effects on farmers’ adoption intensity of LAPs, and the results are robust in different estimations. The results also suggest that the effects of social media and adoption intensity of LAPs on different income levels of farmers are heterogenous. Specifically, adoption intensity of LAPs can significantly increase the household income of farmers with the high-income level. Farmers’ participation in the short-video social media significantly increased household income of farmers with the lower income level.

Our findings highlight the importance of the emerging social media in rural development, and have several policy implications. First, extension agencies can take advantage of the new short-video social media to promote agricultural innovations in rural areas where the smartphone has been widely used. It should not be limited to traditional models of agricultural extension. Policymakers can facilitate extension agencies to deliver useful information about agricultural innovations to farmers through the short-videos. For example, more budget can be used for making demonstration short videos and help smallholder farmers access Internet economically. Second, policymakers can provide trainings for smallholder farmers to make better use of the short-video social media. The social media is an important channel to learn and spread the innovative agricultural practices for farmers who are even illiterate. Policymakers also can encourage well-educated farmers to play a leading role in diffusion of new agricultural practices among farmers trough releasing short videos on the social media. Third, the incentive measures should be designed for smallholder farmers to strengthen the adoption of LAPs and increase their incomes at all levels.

Though this study makes a marginal contribution to the existing literature about the use of LAPs in agriculture, it still has limitations. First, our findings can be restricted to particular regions where the short-video social media becomes popular. The conclusions should be cautiously extended to other cases. Second, we perform the analysis with cross-sectional data, while the economic benefits of LAPs are more observable after a period of time. Thus, future studies could be conducted with a panel dataset. Third, because each LAP may exert a different impact on farmers’ incomes, the estimation based on adoption intensity may lead to the problem of information loss. Other methods (i.e., multinomial endogenous switching regression model) can be used to address this issue in the future studies.

## Data Availability Statement

The raw data supporting the conclusions of this article will be made available by the authors, without undue reservation.

## Ethics Statement

Ethical review and approval was not required for the study on human participants in accordance with the local legislation and institutional requirements. Written informed consent for participation was not required for this study in accordance with the national legislation and the institutional requirements.

## Author Contributions

QY: conceptualization, methodology, investigation, formal analysis, and writing — original draft. YZ: conceptualization, investigation, formal analysis, project administration, and writing — review and editing. FW: investigation and funding acquisition. All authors contributed to the article and approved the submitted version.

## Conflict of Interest

The authors declare that the research was conducted in the absence of any commercial or financial relationships that could be construed as a potential conflict of interest.

## Publisher’s Note

All claims expressed in this article are solely those of the authors and do not necessarily represent those of their affiliated organizations, or those of the publisher, the editors and the reviewers. Any product that may be evaluated in this article, or claim that may be made by its manufacturer, is not guaranteed or endorsed by the publisher.
